# Supplementary material for: Call combinations and compositional processing in wild chimpanzees
Source: Nat Commun. 2023 May 4;14:2225. doi: 10.1038/s41467-023-37816-y (PMC10160036; doi:10.1038/s41467-023-37816-y)
Supplement: Supplementary file 1 — Supplementary Information [file 41467_2023_37816_MOESM1_ESM.pdf]

## Supplementary Information

## Table of content:


Supplementary Note 1: Natural observations 2

Figure S1: Spectrograms 3

Table S1: Bayesian analyses of looking duration 4

Figure S2: Comparison Bayesian/Frequentist approaches: looking duration 4

Table S2: Bayesian analyses of latency to look 5

Figure S3: Comparison Bayesian/Frequentist approaches: latency to look 5

Table S3: Bayesian analyses of number of looks 6

Figure S4: Comparison Bayesian/Frequentist approaches: number of looks 6

Table S4: List of individuals 7

Figure S5: Snake model – python 8

Figure S6: Snake model – viper 8

Supplementary references 9

**Supplementary Note 1: Natural observations of the production of the "*alarm-huu+waa-bark*" combination**

23.07.18

*Caller*: Heri (subadult female).

*Party composition*: Anna, Cyndi, Hadue, Harmoni, Harriet, Heri, Irene, Ishe, Kalema, Kaija, Kathy, Kefa, Kirabo, Klauce, Kwera, Kutu, Mbotella, Melissa, Muhumuza, Oakland, Ozzy, Sharlot, Upesi.

*Event*: On the 23rd of July 2018, M.L. and B.C. followed a party of 23 individuals feeding on a large Ficus tree (*Ficus mucoso*). At 11:56, Heri, resting in another tree approximately 50 meters away from the Ficus tree, started producing "*alarm-huu+waa-bark*" combinations while looking down towards the ground. At this moment, all 22 individuals remaining on the Ficus tree stopped feeding and started climbing down. All individuals joined Heri on the tree she was in or on nearby smaller trees. None of the individuals remained on the ground. The party left the location at 1240 hours. The snake was not directly observed by humans.

11.06.19

*Caller*: Pascal (adult male).

*Party composition*: Cyndi, Deli, Eve, Hawa, Irene, Ishe, Janie, Kato, Mbotella, Musa, Nambi, Pascal, Zed.

*Event*: On the 11th of June 2019, M.L. and B.C. followed a party of 13 individuals. At approximately 1400 hours, while individuals were resting over a dispersed area, M.L. and B.C. heard "*alarm-huu+waa-bark*" combinations produced by Pascal approximately 30 meters away. After the production of these call combinations, all individuals interrupted resting, stood up and joined Pascal. A pale green snake was seen moving away, suspected to be a Jameson mamba (*Dendroaspis jamesoni*). Subjects resumed resting after approximately 30 minutes.

## Supplementary Figures and Tables

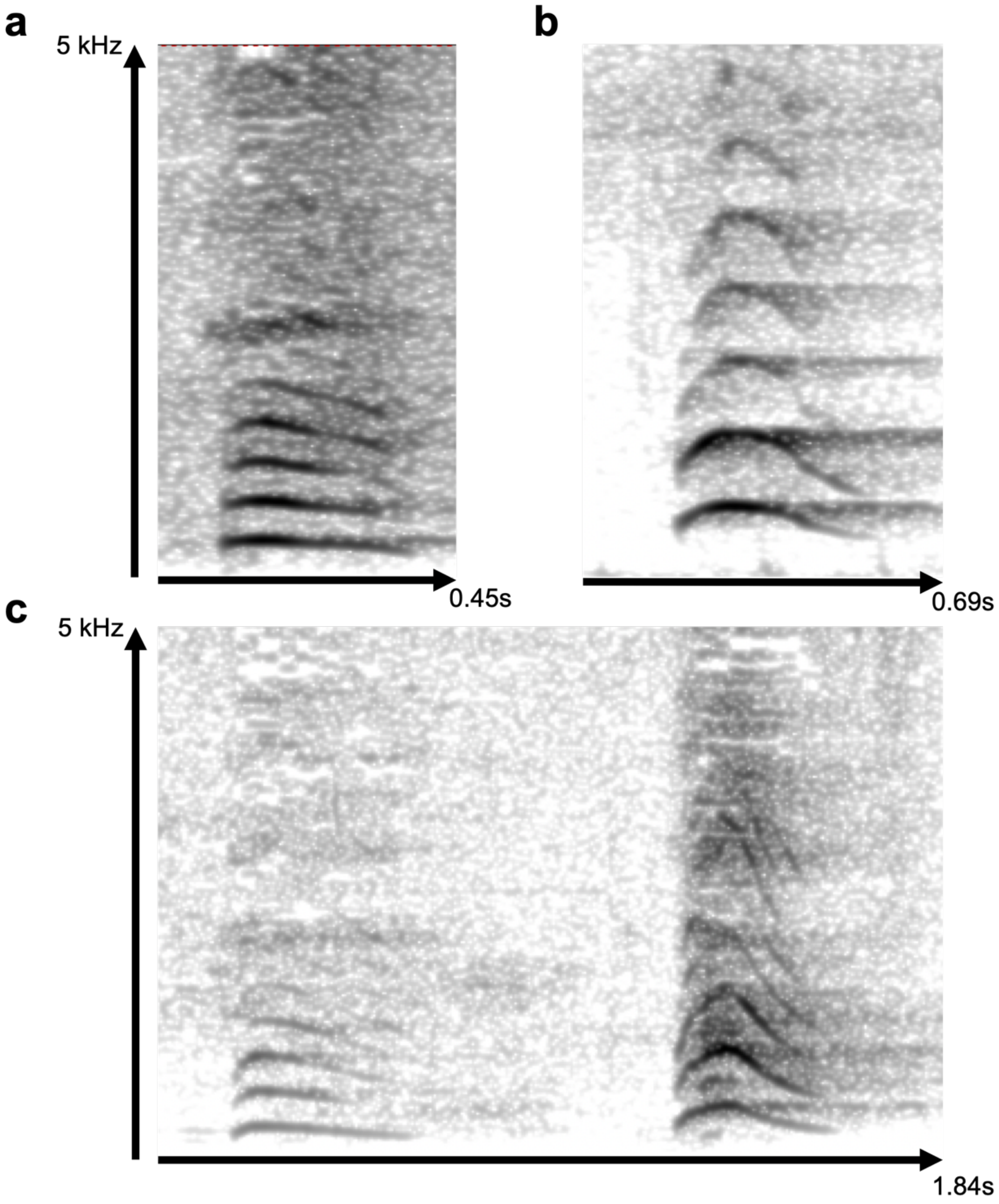


**Fig. S1**. Spectrograms of (a) an "*alarm-huu*", (b) a "*waa-bark*" and (c) an "*alarm-hoo+waa-bark*" combination, produced by an adult male.

**Table S1**. Bayesian analyses of looking duration. Bayesian analyses based on multiple imputation indicating the influence of playback condition on the looking duration towards the loudspeaker. AH: singly-occurring "alarm-huu"; WB: singly-occurring "waa-bark"; AH-WB: "alarm-huu+waa-bark" combination. Estimate: mean of the posterior distribution; Est.Error: standard deviation of the posterior distribution; l-95% CI: 95% credible interval, lower limit; u-95% CI: 95% credible interval, upper limit; Rhat: potential scale reduction statistic; Bulk_ESS: bulk effective sample size; Tail_ESS: tail effective sample size. Biological significance was attributed when credibility intervals did not include zero (bold).

| | Estimate | Est.Error | l-95% CI | u-95% CI | Rhat | Bulk_ESS | Tail_ESS |
|---|---|---|---|---|---|---|---|
| AH / WB | -0.58 | 0.56 | -1.68 | 0.54 | 1.17 | 764 | 1569 |
| AH / AH-WB | -1.7 | 0.5 | **-2.66** | **-0.69** | 1.03 | 3911 | 9246 |
| WB / AH-WB | -1.12 | 0.55 | **-2.22** | **-0.04** | 1.18 | 718 | 2007 |

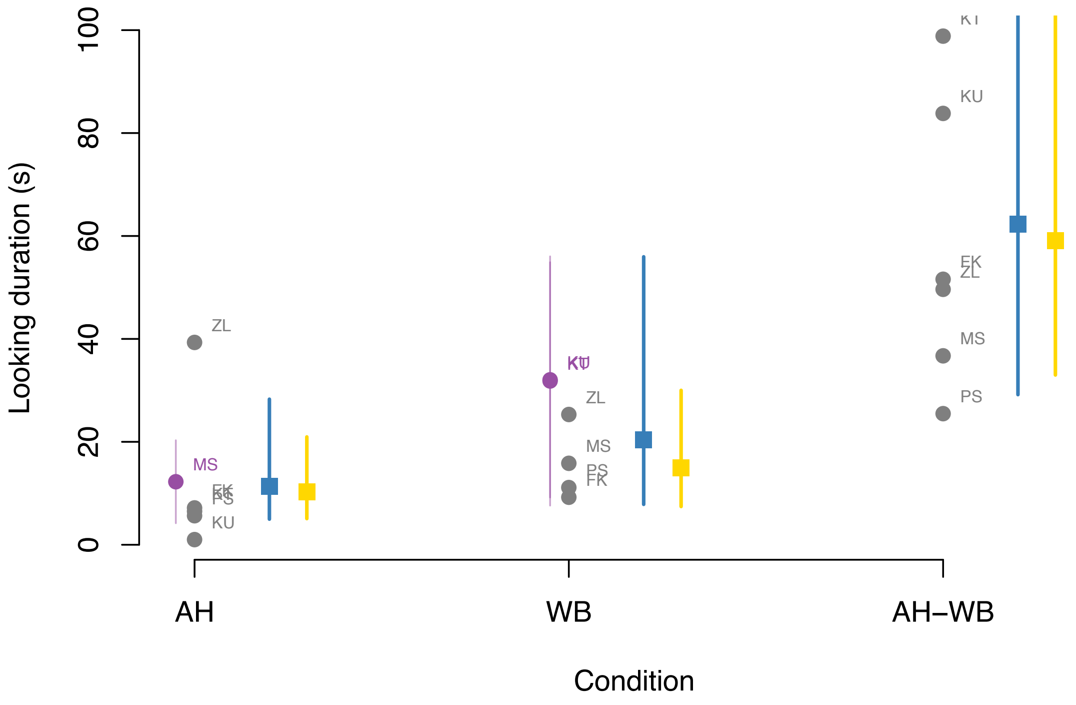


**Fig. S2**. Comparison between GLMM implemented under a Bayesian (blue) vs. frequentist (yellow) framework on the influence of playback condition on the overall looking duration towards the speaker. AH: singly-occurring "alarm-huu" (n=5); WB: singly-occurring "waa-bark" (n=4); AH-WB: "alarm-huu+waa-bark" combination (n=6). Grey dots indicate the raw data, purple dots and error bars indicate the multiple imputations for the three missing data points (mean ± standard deviation), blue squares and error bars indicate model estimates using a Bayesian approach (mean ± standard deviation), yellow squares and error bars represent model estimates presented in the main text (mean ± standard deviation) (frequentist approach).

**Table S2**. Bayesian analyses of latency to look. Bayesian analyses based on multiple imputation indicating the influence of playback condition on the latency to look towards the loudspeaker. AH: singly-occurring "alarm-huu"; WB: singly-occurring "waa-bark"; AH-WB: "alarm-huu+waa-bark" combination. Estimate: mean of the posterior distribution; Est.Error: standard deviation of the posterior distribution; l-95% CI: 95% credible interval, lower limit; u-95% CI: 95% credible interval, upper limit; Rhat: potential scale reduction statistic; Bulk_ESS: bulk effective sample size; Tail_ESS: tail effective sample size. Biological significance was attributed when credibility intervals did not include zero (bold).

| | Estimate | Est.Error | l-95% CI | u-95% CI | Rhat | Bulk_ESS | Tail_ESS |
|---|---|---|---|---|---|---|---|
| AH / WB | 2.37 | 1.03 | **0.08** | **4.15** | 1.27 | 536 | 1077 |
| AH / AH-WB | 2.95 | 0.85 | **1.17** | **4.56** | 1.07 | 1737 | 2994 |
| WB / AH-WB | 0.58 | 0.89 | -1.01 | 2.56 | 1.15 | 823 | 1305 |

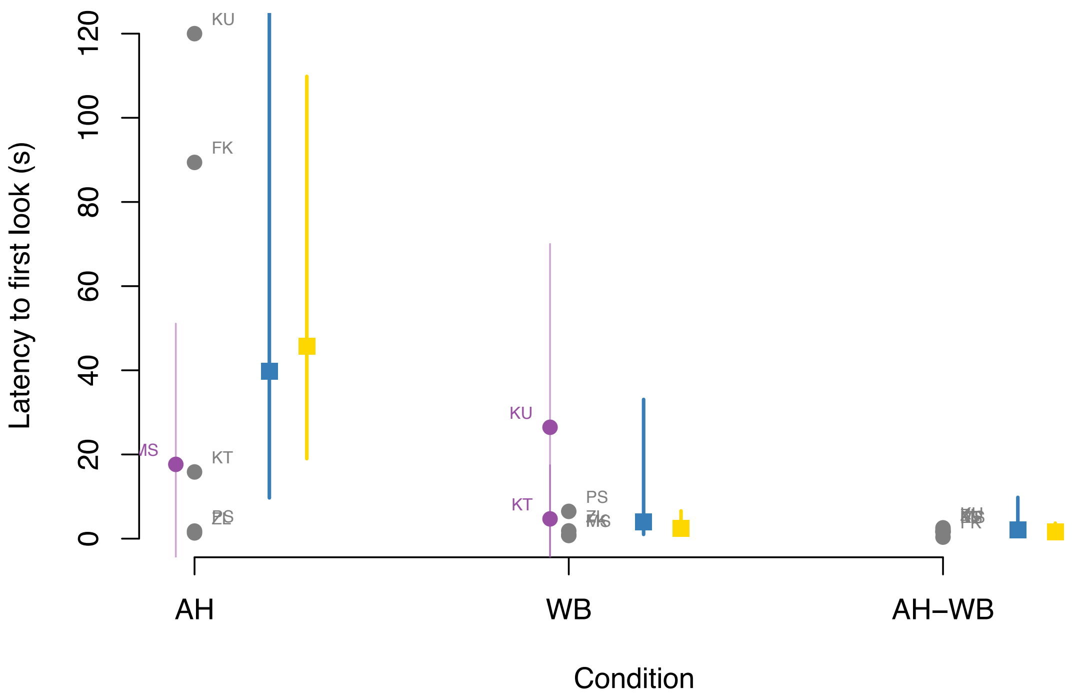


**Fig. S3**. Comparison between GLMM implemented under a Bayesian (blue) vs. frequentist (yellow) framework on the influence of playback condition on the latency to look at the speaker. AH: singly-occurring "alarm-huu" (n=5); WB: singly-occurring "waa-bark" (n=4); AH-WB: "alarm-huu+waa-bark" combination (n=6). Grey dots indicate the raw data, purple dots and error bars indicate the multiple imputations for the three missing data points (mean ± standard deviation), blue squares and error bars indicate model estimates using a Bayesian approach (mean ± standard deviation), yellow squares and error bars represent model estimates presented in the main text (mean ± standard deviation) (frequentist approach).

**Table S3**. Bayesian analyses of number of looks. Bayesian analyses based on multiple imputation indicating the influence of playback condition on the number of looks towards the loudspeaker. AH: singly-occurring “alarm-huu”; WB: singly-occurring “waa-bark”; AH-WB: “alarm-huu+waa-bark” combination. Estimate: mean of the posterior distribution; Est.Error: standard deviation of the posterior distribution; l-95% CI: 95% credible interval, lower limit; u-95% CI: 95% credible interval, upper limit; Rhat: potential scale reduction statistic; Bulk_ESS: bulk effective sample size; Tail_ESS: tail effective sample size.

| | Estimate | Est.Error | l-95% CI | u-95% CI | Rhat | Bulk_ESS | Tail_ESS |
|---|---|---|---|---|---|---|---|
| AH / WB | -0.31 | 0.45 | -1.21 | 0.57 | 1.14 | 893 | 4051 |
| AH / AH-WB | -0.61 | 0.41 | -1.42 | 0.18 | 1.06 | 1888 | 10017 |
| WB / AH-WB | -0.29 | 0.39 | -1.07 | 0.47 | 1.08 | 1501 | 5991 |

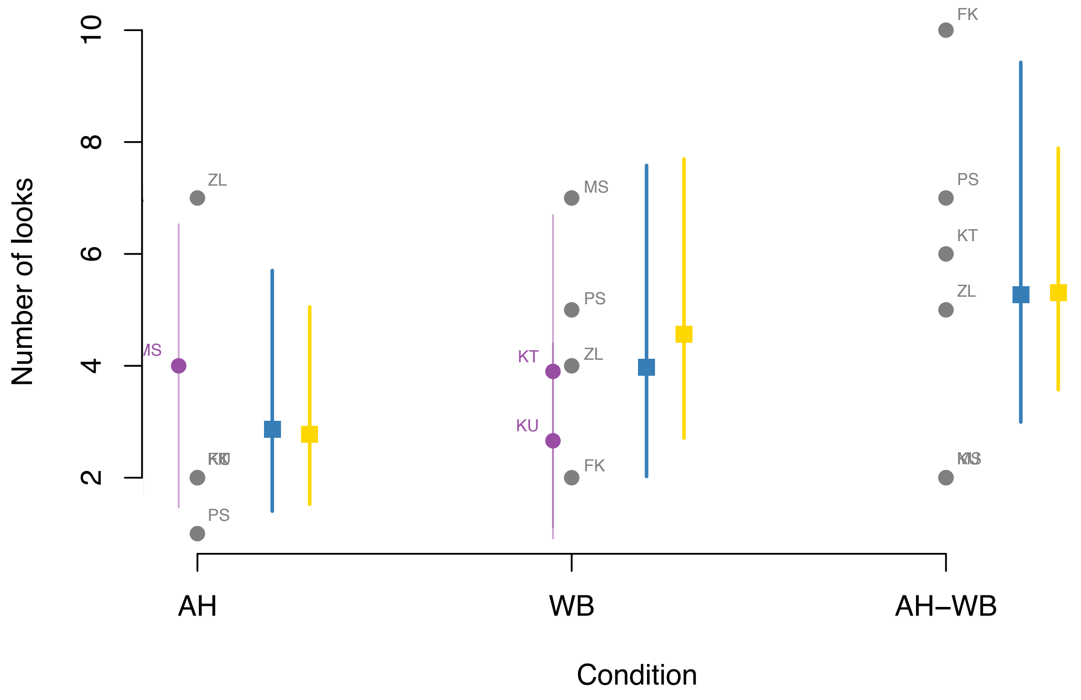


**Fig. S4**. Comparison between GLMM implemented under a Bayesian (blue) vs. frequentist (yellow) framework on the influence of playback condition on number of looks towards the speaker. AH: singly-occurring “alarm-huu” (n=5); WB: singly-occurring “waa-bark” (n=4); AH-WB: “alarm-huu+waa-bark” combination (n=6). Grey dots indicate the raw data, purple dots and bars indicate the multiple imputations for the three missing data points (mean ± standard deviation), blue squares and bars indicate model estimates using a Bayesian approach (mean ± standard deviation), yellow squares and bars represent model estimates presented in the main text (mean ± standard deviation) (frequentist approach).

**Table S4**. List of chimpanzees from the Sonso community.

| Name ID | Sex | Age |
|---|---|---|
| Alice | Female | Infant |
| Anna | Female | Adult |
| Cyndi | Female | Adult |
| Deli | Female | Adult |
| Dembe | Female | Infant |
| Dora | Female | Adult |
| Eve | Female | Adult |
| Faida | Female | Subadult |
| Faith | Female | Juvenile |
| Flora | Female | Adult |
| Frank | Male | Adult |
| Geoffrey | Male | Juvenile |
| Gladys | Female | Adult |
| Goria | Female | Subadult |
| Hadue | Male | Infant |
| Harmoni | Female | Juvenile |
| Harriet | Female | Adult |
| Hawa | Male | Adult |
| Heri | Female | Subadult |
| Irene | Female | Adult |
| Ishe | Female | Infant |
| Jacintha | Female | Infant |
| Jacob | Male | Juvenile |
| James | Male | Subadult |
| Janie | Female | Adult |
| Juliet | Female | Adult |
| Kaija | Male | Juvenile |
| Kalema | Female | Adult |
| Kaqwa | Male | Infant |
| Kasigwa | Male | Adult |
| Kaspa | Female | Subadult |
| Kathy | Female | Subadult |
| Katia | Female | Adult |
| Kato | Male | Adult |
| Kavera | Male | Juvenile |
| Kefa | Male | Juvenile |
| Kewaya | Female | Adult |
| Kigere | Female | Adult |
| Kirabo | Male | Juvenile |
| Klauce | Male | Subadult |
| Kox | Female | Subadult |
| Kutu | Female | Adult |
| Kwera | Female | Adult |
| Kwezy[Δ] | Male | Adult |
| Linda | Female | Adult |
| Marion | Female | Adult |
| Mbotella | Male | Juvenile |
| Melissa | Female | Adult |
| Muhumuza | Male | Infant |
| Mukwano | Female | Adult |
| Musa | Male | Adult |
| Nambi | Female | Adult |
| Oakland | Female | Adult |
| Ozzie | Male | Juvenile |
| Pascal | Male | Adult |
| Rafia | Female | Subadult |
| Ramula | Female | Adult |
| Roman | Male | Infant |
| Rose | Female | Adult |
| Ruhara | Female | Adult |
| Sharlot | Female | Subadult |
| Simon | Male | Adult |
| Squibs[Δ] | Male | Adult |
| Tanja | Female | Adult |
| Twenty | Female | Subadult |
| Upesi | Female | Adult |
| Waseme | Female | Adult |
| Yuliyo | Female | Adult |
| Zalu | Male | Adult |
| Zambe | Female | Adult |
| Zed | Male | Adult |
| Zefa[Δ] | Male | Adult |

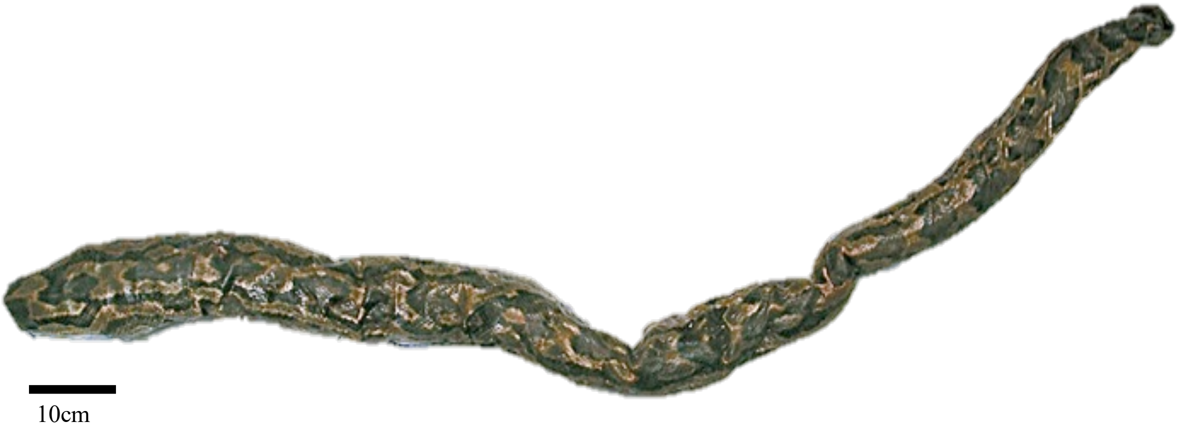


**Fig. S5.** Snake model presented in between 2010 and 2011 for the snake presentation experiments (adapted from Schel et al., 2013).

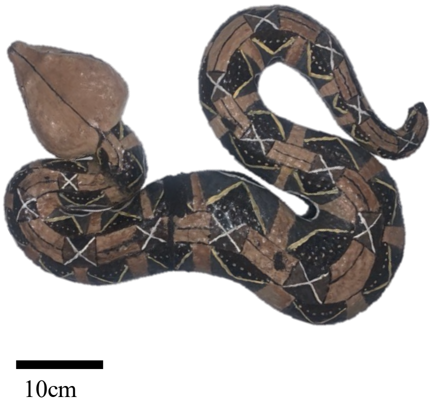


**Fig. S6.** Snake model presented in 2019 to accrue a satisfactory number of playback stimuli.

**Supplementary References**

Schel, A. M. *et al.* Chimpanzee Alarm Call Production Meets Key Criteria for Intentionality. *PLoS One* **8**, e76674 (2013).
