# Supplementary material for: Call combinations and compositional processing in wild chimpanzees
Source: Nat Commun. 2023 May 4;14:2225. doi: 10.1038/s41467-023-37816-y (PMC10160036; doi:10.1038/s41467-023-37816-y)
Supplement: Supplementary file 3 — Description of Additional Supplementary Files [file 41467_2023_37816_MOESM3_ESM.pdf]

Description of Additional Supplementary Files

File Name: Supplementary Movie 1
Description: Video illustrating a reaction to the playback of an “alarm-huu+waa-bark” combination. Focal male KT looks in the direction of the loudspeaker, stands up and moves towards the loudspeaker, and produces a pant-hoot accompanied by a display towards the loudspeaker before climbing a tree briefly at approximately 1m height. Available at https://github.com/MaelLeroux/AHWB/tree/Video
