# Supplementary material for: Call combinations and compositional processing in wild chimpanzees
Source: Nat Commun. 2023 May 4;14:2225. doi: 10.1038/s41467-023-37816-y (PMC10160036; doi:10.1038/s41467-023-37816-y)
Supplement: Supplementary file 5 — Reporting Summary [file 41467_2023_37816_MOESM5_ESM.pdf]

nature portfolio

Corresponding author(s): Mael Leroux

Last updated by author(s): Mar 28, 2023

Reporting Summary

Nature Portfolio wishes to improve the reproducibility of the work that we publish. This form provides structure for consistency and transparency in reporting. For further information on Nature Portfolio policies, see our Editorial Policies and the Editorial Policy Checklist.

Statistics

For all statistical analyses, confirm that the following items are present in the figure legend, table legend, main text, or Methods section.

n/a	Confirmed	
☐	☒	The exact sample size (*n*) for each experimental group/condition, given as a discrete number and unit of measurement
☐	☒	A statement on whether measurements were taken from distinct samples or whether the same sample was measured repeatedly
☐	☒	The statistical test(s) used AND whether they are one- or two-sided<br>*Only common tests should be described solely by name; describe more complex techniques in the Methods section.*
☐	☒	A description of all covariates tested
☐	☒	A description of any assumptions or corrections, such as tests of normality and adjustment for multiple comparisons
☐	☒	A full description of the statistical parameters including central tendency (e.g. means) or other basic estimates (e.g. regression coefficient) AND variation (e.g. standard deviation) or associated estimates of uncertainty (e.g. confidence intervals)
☐	☒	For null hypothesis testing, the test statistic (e.g. *F*, *t*, *r*) with confidence intervals, effect sizes, degrees of freedom and *P* value noted<br>*Give P values as exact values whenever suitable.*
☐	☒	For Bayesian analysis, information on the choice of priors and Markov chain Monte Carlo settings
☒	☐	For hierarchical and complex designs, identification of the appropriate level for tests and full reporting of outcomes
☒	☐	Estimates of effect sizes (e.g. Cohen's *d*, Pearson's *r*), indicating how they were calculated

Our web collection on statistics for biologists contains articles on many of the points above.

Software and code

Policy information about availability of computer code

Data collection	No software was used for data collection
Data analysis	We used Boris, v.8.5 to code videos and R, v.2022.02.3 for statistical analyses. Specifically, we used the packages lme4, glmmTMB, mice, brms, DHARMa. The code that supports the findings of this study are publicly available online at https://github.com/MaelLeroux/AH-WB

For manuscripts utilizing custom algorithms or software that are central to the research but not yet described in published literature, software must be made available to editors and reviewers. We strongly encourage code deposition in a community repository (e.g. GitHub). See the Nature Portfolio guidelines for submitting code & software for further information.

Data

Policy information about availability of data

All manuscripts must include a data availability statement. This statement should provide the following information, where applicable:

- Accession codes, unique identifiers, or web links for publicly available datasets
- A description of any restrictions on data availability
- For clinical datasets or third party data, please ensure that the statement adheres to our policy

All data supporting the findings of this study are publicly available at https://github.com/MaelLeroux/AH-WB

Human research participants

Policy information about studies involving human research participants and Sex and Gender in Research.

Reporting on sex and gender	The study was not conducted on humans.
Population characteristics	The study was not conducted on humans.
Recruitment	The study was not conducted on humans.
Ethics oversight	The study was not conducted on humans.

Note that full information on the approval of the study protocol must also be provided in the manuscript.

Field-specific reporting

Please select the one below that is the best fit for your research. If you are not sure, read the appropriate sections before making your selection.

☐ Life sciences ☐ Behavioural & social sciences ☒ Ecological, evolutionary & environmental sciences

For a reference copy of the document with all sections, see nature.com/documents/nr-reporting-summary-flat.pdf

Ecological, evolutionary & environmental sciences study design

All studies must disclose on these points even when the disclosure is negative.

Study description

We i) analysed snake presentations conducted for another study (see Schel et al., 2013) and ii) conducted playback experiments consisting on 3 different conditions presented randomly to 6 different subjects.

Research sample

We studied chimpanzees from the Budongo Conservation Field Station, Uganda.
Snake presentations were conducted between January 2010 and December 2011. In total, 13 individuals were exposed to the snake in different spatial constellations, totaling 27 trials (eight males and five females, see Schel et al., 2013 for more details).
Playback experiments were conducted between February 2019 and March 2022 for a total of 14 months. Six well-habituated adult individuals that could be routinely followed when alone were tested as subjects in playback experiments (one female: KU and five males: FK, KT, MS, PS, ZL). In total, we conducted 15 trials on 6 different individuals .

Sampling strategy

To reduce to a minimum the impact on animals, we applied the three Rs principle. Therefore, to investigate the production of the “alarm-hoo+waa-bark” call combination, we analyzed snake presentation experiments conducted in 2010-11 for another study within the Sonso community (See Schel et al., 2013).
We conducted playback experiments on six adult individuals only – i.e. minimum sample size to detect a significant effect, and reduced the number of conditions to the bare minimum (i.e. three) to investigate whether receivers perceived the “alarm-hoo+waa-bark” call combination as a syntactic-like structure.

Data collection

Snake presentations
When a chimpanzee started travelling, one experimenter (E1) followed the individual while another experimenter (E2) moved ahead of this individual and hid the snake model under leaves and branches on its anticipated path. E2 then moved away from this location and concealed themselves from the arriving chimpanzee. When the subject approached the location of the snake model, E2 pulled the string attached to the snake model causing its immediate detection by the subject. E1 then monitored the reaction of the subject. See (Schel et al., 2013) for more details on the procedure.

Playback experiments
All playback stimuli were played back in .wav format using a SanDisk Clip Sport Plus (www.westerndigital.com) connected to a Mipro MA-707 loudspeaker (www.mipro.com). To avoid habituation effects, the loudspeaker was concealed in a backpack (with only a hole cut into the fabric at the sound output level to avoid sound distortion) so the chimpanzees never saw the equipment in operation. Video recordings of trials were conducted using a Sony Handycam HDR-CX240 or Panasonic HC-V777 video cameras. The protocol implemented in this study followed a similar methodology validated and used in previous work conducted in the same community of chimpanzees (Slocombe et al., 2009). Specifically, three people were required to run the experiment to ensure aforementioned ethical and realism criteria were met. Specifically, B.C. stayed with the subject who received the playback, M.L. or C.W. played back the stimulus from the loudspeaker at a distance of ~30 m from the subject, and a third person (Denis Lomoro or Jackson Asua) followed the call provider. Contact between the three experimenters was maintained throughout the duration of the experiment using Motorola GP340 radios (www.motorola.com) and Nokia 105 phones (www.nokia.com). Subjects were initially chosen opportunistically, largely determined by which individuals were seen to isolate themselves.
We considered starting an experiment when (i) the subject was observed resting on the ground (to avoid potential bias due to the behavior/position prior to the experiment), (ii) the call provider was at least 300 m away from the subject (to ensure it did not hear its own call being broadcasted) and (iii) no other individuals were present within a 300 m radius (to avoid potential audience effect bias and ensure potential future subjects did not hear the stimulus twice). If these conditions were met, B.C. started filming the subject while M.L. or C.W. took an indirect route to a location of ~30 m from the subject in the direction of the call provider (established through maintaining contact with D.L. or J.A.). The loudspeaker was placed out of sight of the focal subject and oriented

towards it. We played the stimuli only when the subject (i) rested for at least 2 min, (ii) was clearly visible to B.C., and (iii) did not face towards the direction of the loudspeaker, so we could detect any looks oriented towards the loudspeaker. Due to the highly demanding procedure, several trials started without reaching completion and broadcasting of the stimuli, hence representing "mock" experiments allowing us to further reduce the risk of habituation. When all these additional conditions were met, M.L or C.W. played the stimulus and B.C. video-recorded and commented the reaction from the subject, focusing specifically on the looking behavior of the chimpanzee. Finally, once a playback was completed, B.C. and M.L. or C.W. followed the subject for the rest of the day to monitor any mid- to long-term effects on the subject. No indications of stress or fear were observed from the subjects after the experiments or upon reunion with other individuals, including the call provider.

Timing and spatial scale

All data presented in this study were collected within the Sonso community of chimpanzees, Budongo Conservation Field Station, Uganda.

Snake Presentation
Snake presentations were conducted between January 2010 and December 2011.
For the seven focal individuals with repeated exposures to the moving snake, the mean inter-trial duration was 68 days (SD = 49; Range = 15-142 days). This reflected naturalistic chimpanzee-snake encounters, as on average, a snake (i.e. any type of dangerous snake, such as Jameson's mamba, Gabon viper, puff adder, black cobra, spitting cobra, or python) was observed while following the chimpanzees approximately once a month, depending on the area the chimpanzees were ranging in and the season. A further 16 individuals saw the non-moving snake once it had been revealed to the focal and for individuals who saw the snake in multiple trials, on average there were 100 days (SD = 116; range 4-265 days) between exposures.

Playback experiments
Playback experiments were conducted between February 2019 and March 2022.
To avoid overexposure for chimpanzees and limit the risk of habituation, we waited a minimum of one day in between two trials from different subjects (average 27 days, range 1-104 days) and at least a week in between two trials with the same subject (average 90 days, range 28-244 days). Furthermore, we ensured that over the whole study period, no chimpanzee heard the same stimulus twice.

Data exclusions

Since snake presentations were conducted for another study, we excluded trials for which we could not confidently document the variables of interest for this specific study, resulting in the analysis of 21/27 trials.
We did not exclude trials from playback experiments.

Reproducibility

We describe the methodology in details to ensure these experiments can be reproduced.

Randomization

For both snake presentations and playback experiments, trials across individuals were counterbalanced, ensuring subjects did not receive conditions in the same order, thus controlling for order effects.

Blinding

To ensure the coding of the videos was reliable, C.W. blind coded a randomly chosen sample of 5 trials (33%), which were also coded by M.L. The mean intra-class correlation coefficient for the three variables outlined above was found to be 1.00, indicating excellent levels of coder agreement.

Did the study involve field work? ☒ Yes ☐ No

Field work, collection and transport

Field conditions

Field work was conducted in the Budongo Conservation Field Station, Budongo forest, Uganda.
The Budongo Forest is located near Lake Albert in Western Uganda and comprise 428 km2 of semi-deciduous, tropical forest.

Location

1°35' and 1°55' N and 31°08' and 31°42' E

Access & import/export

We collected data at the BCFS, a research field station established in Uganda since 1990. The station provided sensible research and logistical support so the study was conducted in the most responsible and ethical manner possible, for the animals but also the local population. The study was conducted in compliance with local, national and international laws. Furthermore, the study was approved by the Uganda Wildlife Authority (permit number: COD/96/05) and the Uganda National Council for Science and Technology (permit number: NS47ES), Uganda, prior to its start (April 2018), and ethical permission was received from the Animal Welfare & Ethical Review Body from the University of Warwick, United Kingdom (permit number: AWERB.35/18-19 and AWERB.01/19-20).

Disturbance

The experiments conducted in this study caused little, short term disturbances. This was minimized by i) mining existing data and ii) conducting the very minimum number of trials necessary. Furthermore, once a trial was completed, we followed the subject for the rest of the day to monitor any mid- to long-term effects on the subject. No indications of stress or fear were observed from the subjects after the experiments or upon reunion with other individuals, including the call provider.

Reporting for specific materials, systems and methods

We require information from authors about some types of materials, experimental systems and methods used in many studies. Here, indicate whether each material, system or method listed is relevant to your study. If you are not sure if a list item applies to your research, read the appropriate section before selecting a response.

Materials & experimental systems

n/a	Involved in the study
☒	☐ Antibodies
☒	☐ Eukaryotic cell lines
☒	☐ Palaeontology and archaeology
☐	☒ Animals and other organisms
☒	☐ Clinical data
☒	☐ Dual use research of concern

Methods

n/a	Involved in the study
☒	☐ ChIP-seq
☒	☐ Flow cytometry
☒	☐ MRI-based neuroimaging

Animals and other research organisms

Policy information about studies involving animals; ARRIVE guidelines recommended for reporting animal research, and Sex and Gender in Research

Laboratory animals	The study did not involve laboratory animals
Wild animals	All animals observed were adult chimpanzees from both sexes. The study did not involve any physical manipulations of the animals.
Reporting on sex	Both sexes were investigated in this study.
Field-collected samples	The study did not involve samples collected from the field.
Ethics oversight	Ethical permission to conduct the study was received from the Animal Welfare & Ethical Review Body from the University of Warwick, United Kingdom (permit number: AWERB.35/18-19 and AWERB.01/19-20) and was further approved by the Uganda Wildlife Authority (permit number: COD/96/05) and the Uganda National Council for Science and Technology (permit number: NS47ES), Uganda.

Note that full information on the approval of the study protocol must also be provided in the manuscript.
